# *Even* in presupposition denials

**DOI:** 10.1007/s10988-023-09402-4

**Published:** 2024-10-03

**Authors:** Naomi Francis

**Affiliations:** Department of Linguistics, University of Ottawa, Ottawa, Canada

**Keywords:** Even, Presupposition denial, Additivity, Alternatives

## Abstract

This paper explores a puzzling polarity-based asymmetry in the use of *even* in sentences that deny presuppositions. It argues that this asymmetry is produced by the interaction of *even*’s controversial additive presupposition with the alternatives that are salient in the relevant contexts and demonstrates that this proposal makes good crosslinguistic predictions. Along the way, this paper shows that presupposition denials are a fruitful testing ground for uncovering details about the behaviour of *even* and the role of presuppositions triggered within focus alternatives.

## A puzzle

*Even* can be used in sentences that deny presuppositions. For example, in the dialogue in (1), Speaker A’s question presupposes that Joanna smoked at some time in the past; Speaker B objects to this presupposition with a sentence containing *even*. 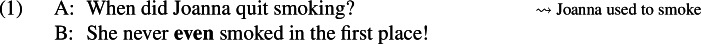


This use of *even* exhibits a curious restriction: it is only acceptable in negative sentences,^1^ as illustrated for a variety of presupposition triggers below.
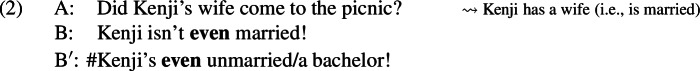


This pattern is surprising, because the positive and negative responses in each example appear to be equivalent in meaning. Moreover, this asymmetry is not reducible to independent properties of *even* or of presupposition denial. Positive sentences with *even* are not generally banned; such sentences are perfectly acceptable when they are not used to deny presuppositions, as (5) demonstrates.
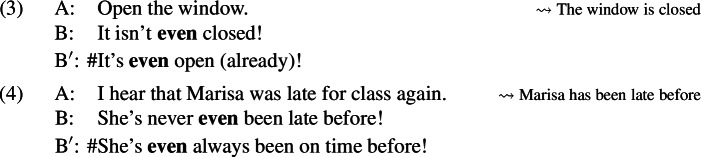


Positive sentences are also not generally incompatible with presupposition denial; if *even* is removed from the sentences above in presupposition-denying contexts, the asymmetry disappears, as illustrated in (6).
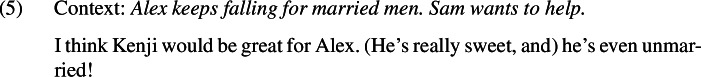


This suggests that the asymmetry reflects something about how *even* and presupposition denial interact. The goal of this paper is to find out what this something is.

The paper is organized as follows: Sect. 2 lays out background assumptions about *even*. Section 3 presents the proposed solution to the puzzle outlined above. This proposal derives the observed asymmetry from the interaction of the additive presupposition of *even* with the focus alternatives that are salient in presupposition-denying discourse contexts. The next two sections defend each of these components in turn: Sect. 4 provides arguments for the particular choice of focus alternatives made in Sect. 3, while Sect. 5 addresses objections that have been raised regarding *even*’s additive presupposition. Section 6 provides crosslinguistic support for the proposal. With this foundation in hand, Sect. 7 discusses a restriction on the position of *even* in negative presupposition denials and argues that it reflects a more general constraint on focus-sensitive operators. Section 8 examines what *even* contributes to those presupposition denials where it is felicitous. Section 9 identifies loose ends and possibilities for extending the proposed analysis. Section 10 concludes.

## Background

*Even* contributes to the meaning of a sentence that hosts it in two ways. For example, the presence of *even* in (7) licenses the inferences in (7-a) and (7-b).
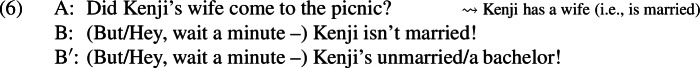


I will assume, following Karttunen and Peters (1979), Rooth (1985), and much subsequent work, that *even* is a focus-sensitive expression with a meaning along the lines of (8).^2^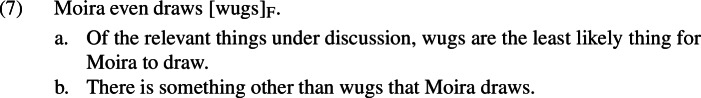 According to this denotation, *even* is a function that takes two arguments: i) a proposition *p*, which corresponds to the prejacent (material in the scope of *even*), and ii) a set of propositions *C*, which corresponds to a contextually salient subset of the focus alternatives. I will assume Rooth’s (1985, 1992) approach to focus, in which alternatives are derived from the prejacent by making substitutions of the appropriate type for the focused constituent.^3^*Even* introduces two presuppositions:^4^ i) a scalar presupposition, which requires that the prejacent is less likely ($<_{\text {w}}$) than any other member of C, and ii) an additive presupposition, which requires that there is at least one member of C other than the prejacent which is true. When defined, *even* is truth-conditionally vacuous; it returns the prejacent unchanged.

To see how this works concretely, let us work through the example in (7). We will assume that the Logical Form (LF) and set of salient alternatives (C) for this sentence are as in (9).
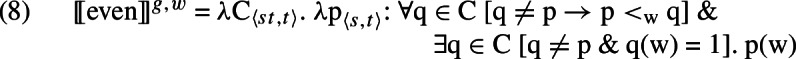


Given the denotation for *even* in (8), this sentence will presuppose that i) it is less likely that Moira draws wugs than that Moira draws cats or that Moira draws giraffes, and that ii) at least one of the non-prejacent alternatives (*Moira draws cats, Moira draws giraffes*) is true. These are equivalent to the inferences identified in (7). When defined, this sentence asserts that Moira draws wugs.

Something more must be said about sentences where *even* is in a downward-entailing environment. The presence of negation in a sentence like (10) does not just alter what is asserted; it also alters the presuppositional inferences that we draw. 
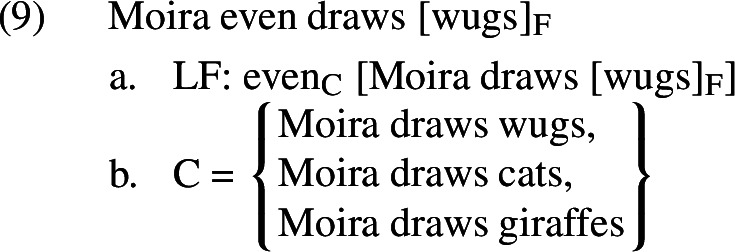


Two kinds of approaches have been proposed to deal with cases like (10), one relying on scope-shifting and the other on lexical ambiguity. The account proposed in this paper will use the scope theory, but for the purposes of future discussion it will be useful to survey both options here.

The ambiguity theory, defended by Rooth (1985), Rullmann (1997), and Erlewine (2018), among others, takes *even* to be lexically ambiguous. On this view, there are two *even*s in English: one with the lexical entry already seen in (8), and one with the lexical entry in (11). The latter differs from the former in the direction of the scalar presupposition and in the truth value required of the non-prejacent alternative by the additive presupposition. The latter *even* is stipulated to be an NPI, meaning that it is restricted to downward-entailing environments.^5^
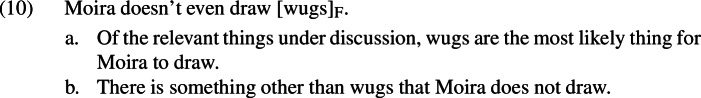


According to the ambiguity theory, the negative sentence in (10) would have an LF and set of alternatives C as in (12). 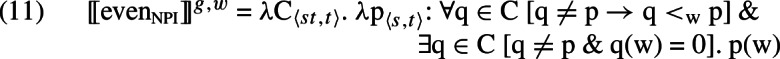 This sentence will be defined if and only if i) it is more likely that Moira draws wugs than that she draws cats or that she draws giraffes, and ii) at least one of the non-prejacent alternatives in C (*Moira draws cats, Moira draws giraffes*) is false. These are equivalent to the inferences in (10). When defined, this sentence asserts that Moira doesn’t draw wugs.

The scope account, defended by Karttunen and Peters (1979), Wilkinson (1996), and Guerzoni (2004), among others, holds that the inferences in (10) are derived by having the *even* defined in (8) take scope above negation at LF, as in (13-a). This produces alternatives that contain negation, as in (13-b).
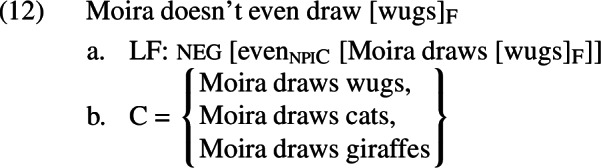


Given this set of alternatives, the sentence in (13) will be defined if and only if i) it is less likely that Moira doesn’t draw wugs than that she doesn’t draw cats or that she doesn’t draw giraffes, and ii) at least one of the non-prejacent alternatives (*Moira doesn’t draw cats, Moira doesn’t draw giraffes*) is true. These are again equivalent to the inferences given in (10). When defined, this sentence asserts that Moira doesn’t draw wugs.

The scope theory and the ambiguity theory are equivalent in their predictions for positive and negative sentences, and so the choice between them does not matter for the cases that this paper addresses. For ease of presentation, I will use the scope theory in the next section. However, nothing hinges on this choice; I sketch the lexical ambiguity counterpart of the analysis proposed here in Appendix A.

## Proposal

To solve the puzzle outlined in Sect. 1, we need to explain the contrast in acceptability between positive and negative presupposition denials with *even*. We also need to explain why this asymmetry is not a general property of sentences with *even* that do not deny presuppositions, or of presupposition denials that do not contain *even*.

Our first task is to decide what is focused in the sentences we are examining. Sentences like the responses in (2) are prosodically ambiguous; as the predicate bears prosodic prominence, the focus associate of *even* must minimally consist of the predicate, but this prosody is also consistent with *even* focusing the entire vP, including the lower copy of the subject. These options are shown schematically in (14).
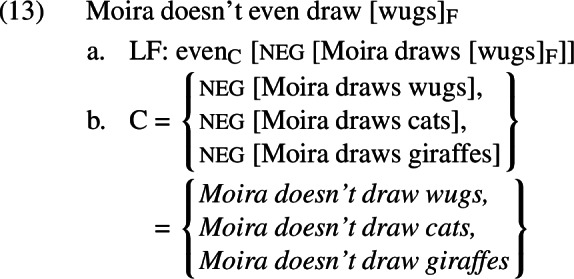


I will assume that *even* associates with the vP as in (14-b)—a propositional constituent (of type <s,t>).^6^ For ease of presentation I will not discuss the option in (14-a) here, but the consequences of choosing this parse are discussed in Appendix B. Assuming vP focus means that the alternatives that *even* makes reference to will be derived from the prejacent by making substitutions for this proposition-sized constituent. In the contexts that we are considering, Speaker A’s discourse move will make certain propositions particularly salient, and thus particularly attractive as substitutions.^7^ For example, a wh-question like (15-a) will make salient the set of possible answers in (15-b) denoted by the question. If Speaker A’s move was to ask a polar question like (16-a), the same logic predicts that the set of propositions in (16-b) will be made salient. Farkas and Bruce (2010) argue that the default responses to a declarative *p* are the same as the default responses to the corresponding polar question *p?*: *yes* and *no*, which are equivalent to asserting *p* and $\lnot$*p*, respectively. I will therefore assume that a declarative like (17-a) makes salient the same propositions as the polar question, as in (17-b).^8^
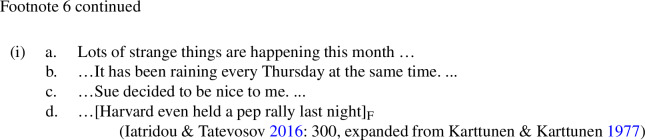


Crucially, these substitutions all contain the trigger for the presupposition that Speaker B denies. This means that the alternatives built from them will presuppose that Kenji has a wife; they will therefore be presupposition failures whenever the prejacent is true, as shown in (18)–(19).
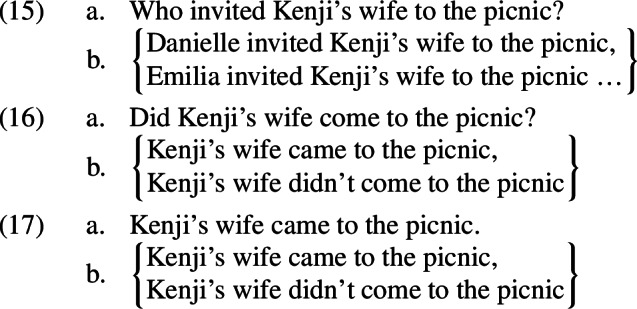


It is not obvious how the scalar presupposition of *even* should be evaluated here, as the non-prejacent alternatives have presuppositions that the prejacent lacks.^9^ The fate of *even*’s additive presupposition, however, is clear. If the non-prejacent alternatives all contain the trigger for the presupposition that the prejacent denies, they will be presupposition failures whenever the prejacent is true. In both the positive and the negative case, this will in turn result in the sentence that is uttered being a presupposition failure due to the additive presupposition of *even*; because all of the relevant non-prejacent alternatives are undefined, none of them can be true.

This cannot be the whole story. Our task is to derive an asymmetry between positive and negative presupposition denials with *even*, and so far we predict that both kinds of sentences will suffer from a failure of the additive presupposition. However, we can now articulate what we need. To explain why the negative presupposition denials with *even* are acceptable, we need to ensure that the additive presupposition of *even* is satisfied in just these cases; this means that we need to find a way for the presupposition-trigger-bearing alternatives that *even* sees to not be presupposition failures just in case they contain negation.

This desideratum dovetails nicely with the second task that we set ourselves at the beginning of this section; we need to explain why our puzzling asymmetry only shows up in presupposition-denying sentences with *even*, and not in sentences with *even* that do not deny presuppositions. I propose that the key lies in a tool that is used to deal with presupposition-denying sentences more generally in trivalent systems.^10^ This tool is Bochvar ’s (1939) so-called meta-assertion operator *A*, which takes a propositional argument and asserts that it is true. The truth table for the A-operator is given in (20). 
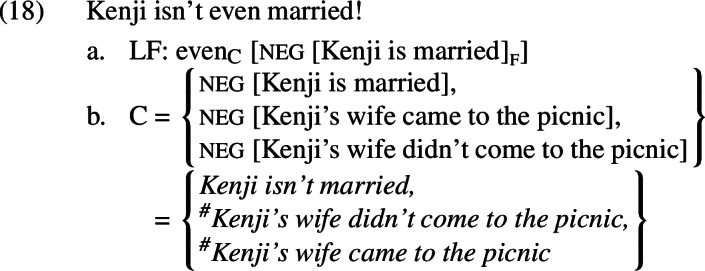


As Beaver and Krahmer (2001) observe, this operator has the effect of converting presuppositions into part of what is asserted. When applied to a proposition that is true or false in the world of evaluation, the A-operator will be vacuous. However, when applied to a proposition that is neither true nor false in the evaluation world, as is the case for a presupposition failure, the A-operator will map this proposition to false; this can be thought of as equivalent to conjoining the presupposition with the asserted meaning of the sentence. Importantly, this operator has been argued to provide a useful way of understanding presupposition denials like (21).^11^
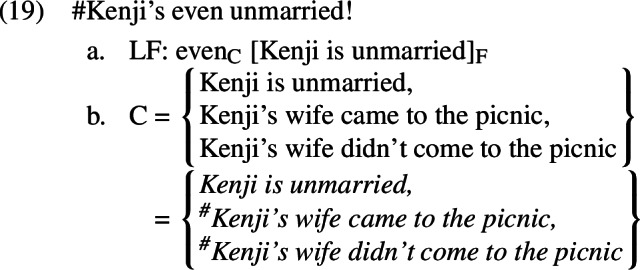


The fact that this sentence is acceptable is surprising. The definite description *the king of France* in the first clause triggers the presupposition that there is a (unique) king of France. Because negation is a hole for presupposition projection (Karttunen 1973), this should become a presupposition of the entire first clause. However, the second clause asserts that there is no king of France. If the second clause is true, the first clause should be a presupposition failure; if the first clause is not a presupposition failure, the second clause should be false. In actual fact, (21) is a perfectly coherent thing to say. Beaver and Krahmer (2001) observe that adopting a parse of the first clause with a silent A-operator below negation allows its presupposition to become part of the asserted content; this allows the presupposition to be negated instead of projected. This in turn makes the first clause compatible with the continuation in the second clause, hence the observed acceptability.
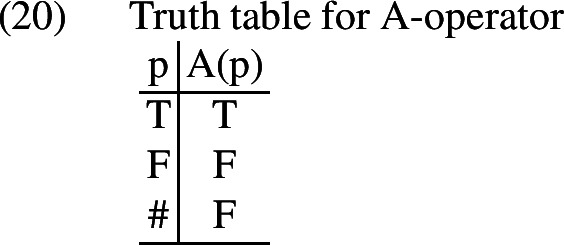


I propose that there is a parse of the presupposition denials with *even* that we are interested in that includes the A-operator, and that this is what accounts for our polarity-based asymmetry. The A-operator only makes it possible to deny presuppositions when it is below negation; let us therefore assume that the relevant parse places the A-operator below negation in these sentences. This will prevent the negative alternatives from being undefined and instead allow them to assert something that is compatible with the prejacent.

Let us see how this works for the negative presupposition denial *Kenji isn’t even married*. If we assume the parse in (23-a), the alternatives will all contain an A-operator under negation, as in (23-b).
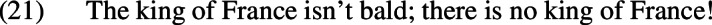


As we have already seen, the material below the A-operator in the non-prejacent alternatives (*Kenji’s wife came to the picnic, Kenji’s wife didn’t come to the picnic*) would be a presupposition failure in the contexts we are considering. The A-operator prevents this presupposition from projecting across negation to turn the entire alternative into a presupposition failure. Instead, it maps *Kenji’s wife came to the picnic* and *Kenji’s wife didn’t come to the picnic* to false.^12^ When negation encounters the result of applying A to *Kenji’s wife came to the picnic* and *Kenji’s wife didn’t come to the picnic* (equivalent to *it’s true that Kenji has a wife and she came to the picnic* and *it’s true that Kenji has a wife and she didn’t come to the picnic*, respectively), the result will be the propositions that it is not true that Kenji has a wife and she came to the picnic and that it is not true that Kenji has a wife and she came to the picnic, respectively. Both propositions are entailed by the prejacent, meaning that whenever the prejacent is true these alternatives are true as well. This trivially satisfies the additive presupposition of *even*. The scalar presupposition is likewise trivially satisfied; because the prejacent asymmetrically entails both of the salient alternatives, it cannot be more likely than them. Thanks to the A-operator and the higher negation in each of the alternatives, the presupposition denial *Kenji isn’t even married!* is no longer a presupposition failure. We thus predict it to be acceptable, as observed.

If this analysis is to be successful, we must ensure that allowing A-operators in our LFs will not lead us to predict that the corresponding positive presupposition denials are acceptable. Let us therefore consider what will happen if we select a parse for *Kenji’s even unmarried* that includes an A-operator, as in (24-a), instead of (19-a).
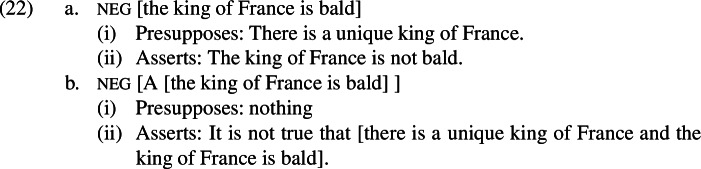


As in the negative case, all of the alternatives will contain the A-operator under this parse; unlike the negative cases, however, they will not contain negation above the A-operator. As in the negative case, applying *A* to *Kenji’s wife didn’t come to the picnic* and *Kenji’s wife came to the picnic* will yield false propositions (equivalent to *it’s true that Kenji has a wife and she didn’t come to the picnic* and *it’s true that Kenji has a wife and she came to the picnic*, respectively). However, because these alternatives contain no negation above the A-operator, they will both remain false. This will result in a failure of the additive presupposition of *even*, just as the *A*-less parse did.^13^

With this, we have successfully derived the asymmetry at the centre of our puzzle using properties of *even* and properties of presupposition denial. *Even* invokes alternatives and triggers an additive presupposition that at least one of these alternatives (besides the prejacent) is true. The alternatives that are salient in presupposition-denying contexts all contain the trigger for the presupposition that the prejacent denies; they cannot be true when the prejacent is true unless they contain an A-operator, which is independently motivated to appear in presupposition denials, under negation. This configuration can be produced in the alternatives for negative presupposition denials but not for positive ones, and so it is only in negative presupposition denials that a failure of *even*’s additive presupposition can be averted.

## The salient alternatives

The proposal made in the previous section relies on a particular set of focus alternatives being salient. In positive presupposition denials, all of the salient non-prejacent alternatives were crucially incompatible with the prejacent, and thus unable to satisfy *even*’s additive presupposition. However, there are many other focus alternatives which could in principle be derived from the prejacent and are not incompatible with it—for example, *the sky is blue, Kenji is Canadian,* and *people attended the picnic*; if any of these alternatives were to be included in C, the additive presupposition would be satisfiable, and we would wrongly predict that positive presupposition denials with *even* can be felicitous. How are we to ensure that C consists of just those alternatives proposed in Sect. 3?

C is a contextually salient subset of the focus alternatives derivable from the prejacent. The prevailing intuition in the literature is that what it means to be a *contextually salient* subset of the focus alternatives is to be anaphoric to, relevant to, or identical to a contextually salient *question* (see Rooth 1992, Roberts 2012, and Beaver et al. 2017, respectively; the latter two works take contextually salient questions to be QUDs). As noted in Sect. 3, the *even*-denials under investigation respond to a preceding discourse move, and this discourse move will constrain what counts as a contextually salient question. In the case of the now-familiar dialogue about Kenji’s wife and the picnic, repeated below in (25), A’s discourse move supplies a polar question. This question is schematized in (26); *q* represents the proposition (presupposed by A’s question) that Kenji has a wife, while ${p }_{q}$ represents the proposition that Kenji’s wife came to the picnic. A’s question presupposes that the context set contains only worlds where Kenji has a wife and partitions these worlds into two cells, the first containing the worlds where Kenji’s wife went to the party ($p _{q}$-worlds) and the second containing the worlds where Kenji’s wife didn’t go to the party ($\lnot p _{q}$-worlds).
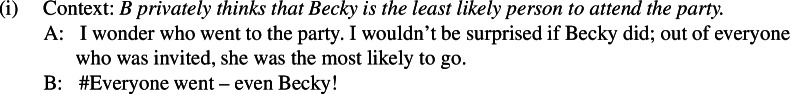


Any proposition that fails to provide a (partial) answer to a question (i.e., that crosscuts the cells of the partition) will be irrelevant to it (Roberts 2012; see also Groenendijk & Stokhof 1984). This is why propositions like *the sky is blue, Kenji is Canadian*, and *people attended the picnic* are excluded from C; they are irrelevant to the salient question.

However, it is worth noting that the prejacent of *even* in this case is not a congruent answer to A’s question; rather than picking out one of the cells in the partition, the prejacent of *even* in both B and $\text {B}^{\prime }$ denotes the set of worlds where Kenji does not have a wife, as indicated by the shading in (27)—a set of worlds that A’s question excluded due to its presupposition.^14^
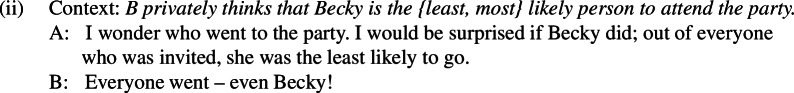


At the same time, this proposition, unlike the irrelevant propositions discussed above, does not crosscut the cells of the partition provided by A’s question. Moreover, it is not the case that the B/$\text {B}^{\prime }$ responses ignore this partition; the non-prejacent alternatives that we have assumed *even* accesses in B are as in (28), and those that *even* accesses in $\text {B}^{\prime }$ are as in (29). Both make use of the division between $\text {p }_{q}$ and $\lnot \text {p }_{q}$ worlds.
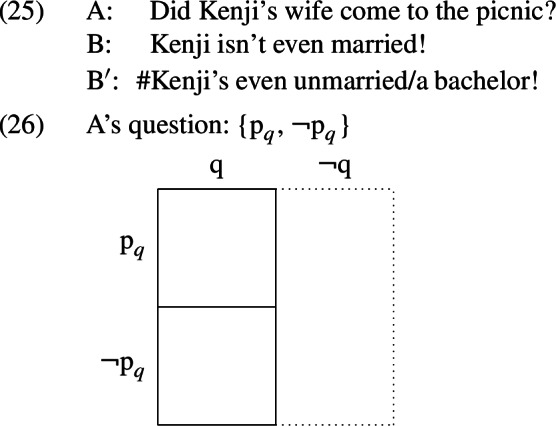


The *even*-sentences that we have been investigating object to a presupposition triggered by a preceding discourse move. It is therefore not surprising that the question made salient by that preceding discourse move does not supply the focus alternatives for *even*. However, while these denials do not directly answer the question raised by the interlocutor, they address a very closely related question — one that differs from the interlocutor’s question just with respect to the worlds excluded by the interlocutor’s presupposition. This new question is still relevant to the initial question, in that its members each rule out at least one cell in the original partition and none of them introduces any new distinctions among worlds. It is this question that corresponds to the value of C.

## The additive component

The proposal made in Sect. 3 relies on the additive presupposition of *even* being unsatisfiable when the salient focus alternatives are incompatible with each other. This presupposition is controversial, and this section examines counterarguments to its existence.

### *Even* with mutually exclusive alternatives

It has been claimed that the additive presupposition of *even* is not active when the alternatives that *even* encounters are mutually exclusive (von Stechow 1991, Krifka 1992, Rullmann 1997, Crnič 2011).^15^

This claim is based on the alleged acceptability of two types of sentences. The first type involves *even* and a lower *only* co-associating with a single element, as in (30). The second type involves alternatives that are mutually exclusive because of independent real-world facts; in (31), the alternatives to *bronze* in the context of medals are *silver* and *gold*, but one cannot win multiple medals for the same event.
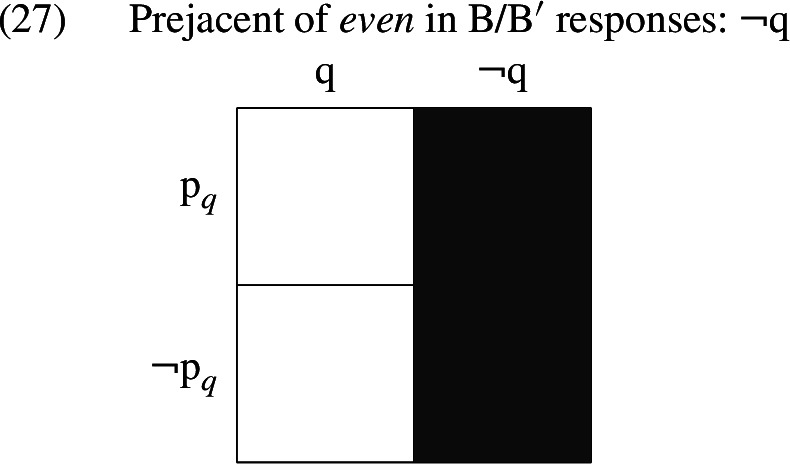


If John only drank water, it cannot be true that he (only) drank anything else. Similarly, if Mary won the silver medal she did not win the bronze medal or the gold medal. If *even* had an additive presupposition, it would not be satisfied in these cases.

To explain the alleged acceptability of these examples, various modifications to the meaning of *even* have been proposed. Crnič (2011) formulates the additive presupposition so that it only applies when the alternatives are not mutually exclusive, while Rullmann (1997) eliminates this presupposition from *even*’s lexical entry entirely, deriving the additive inference instead by pragmatic reasoning. If either of these positions is correct, the analysis proposed above cannot stand; if *even* does not introduce an additive requirement when the alternatives are mutually exclusive, we should predict the positive presupposition denials with *even* to be acceptable, contrary to fact.

Before we reject our analysis, it is worth taking a closer look at the evidence for the claim that *even* is compatible with mutually exclusive alternatives. It turns out that native speakers of English judge the use of *even* in examples like (31) to be unacceptable. This is consistent with *even* having an additive presupposition.^16^ However, native speakers of English do judge (30) to be acceptable.

According to Krifka (1992), (30) is acceptable and conveys that i) John drank water, ii) John did not drink anything other than water, and iii) there are other relevant beverages x such that it is more likely that John drank x and nothing else than that John drank water and nothing else. However, as Wilkinson (1996) observes, the alternatives made salient in the context that Krifka provides are not mutually exclusive propositions of the form *John only drank x*; they are propositions of the form *y only drank x*. This licenses a different parse entirely, where there is a second source of alternatives on the subject.^17^ More particularly, Wilkinson suggests that the subject either bears a free focus or is a second focus-associate of *even*, as indicated in (32). Under this parse, the alternatives available to *even* in (32-b) are no longer mutually exclusive.
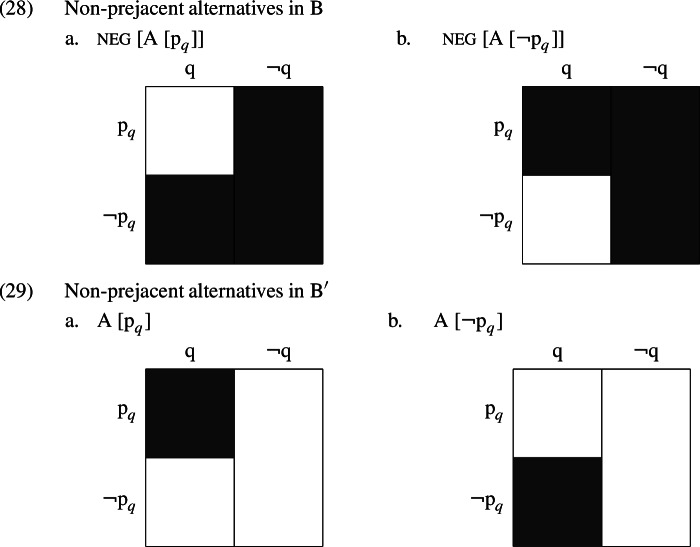


The subject may in fact be a contrastive topic rather than a focus; for arguments that *even* is sensitive to alternatives generated by contrastive topics, see Zimmermann (2015). What matters for our purposes is that the alternatives are allowed to differ from the prejacent in two places, yielding a set of non-mutually-exclusive alternatives as in (32-b). Importantly, speakers report a contrast between (31) and (33), where the context makes salient a set of alternatives that are truly mutually exclusive.^18^ I have been unable to find any speakers who accept (33).
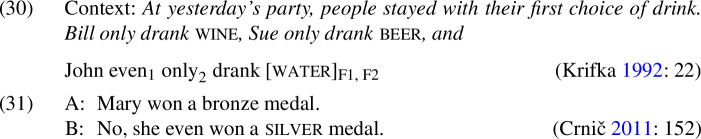


In this subsection, we have seen that the data presented in support of the additive presupposition being inactive when alternatives are mutually exclusive does not show what it was claimed to show. With careful consideration of the contexts involved, it appears that—at least for the speakers I have interviewed—these examples are unacceptable when the salient alternatives are mutually exclusive, but acceptable under a parse that makes them not mutually exclusive.^19^ This is exactly what we should expect if the additive presupposition is active. Thus, these data turn out to be an argument in favour of the additive presupposition being active rather than against it.^20^

### *Even* with non-mutually-exclusive alternatives

A reviewer suggests that the felicity of examples like (34) presents a challenge to the additive view of *even*.^21^
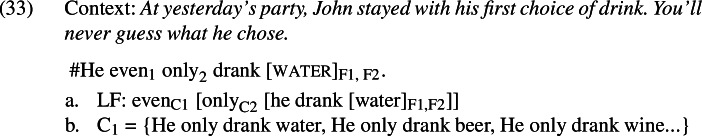


The presence of narrow focus on the subject ensures that *even* considers alternatives of the form *x passed the test*. These alternatives are not mutually exclusive. The context is intended to supply the necessary information to satisfy *even*’s scalar presupposition: if Alex is the worst student in the class, it is plausible that Alex will be the least likely of the students to pass any given test. At the same time, the continuation *How come everyone else failed?* requires that Alex is the only student who passed; this is incompatible with *even*’s additive presupposition. If this example is felicitous, we would need to explain why the additive presupposition apparently has no effect here.

Not all native speakers agree with the reviewer’s judgement for this example; a representative comment offered by one native speaker consultant was “It sounds good until I think about what it means”. One plausible explanation for this intuition lies with the order in which the relevant information is presented. Because the initial context in (34) is compatible with the additive presupposition of *even*, it is only upon hearing the continuation *How come everyone else failed?* that the satisfaction of the additive presupposition is ruled out. Thus, a hearer could initially accommodate the additive presupposition of *even* and judge the *even* sentence to be acceptable, and then have difficulty revising this evaluation in light of the continuation. If this is what is responsible for judgements of acceptability for (34), we should predict that introducing the information that renders the additive presupposition unsatisfied before the sentence containing *even* is uttered will render the example unacceptable. As indicated in (35), this prediction is borne out; even those speakers who accepted (34) find (35) to be infelicitous.
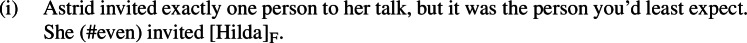


However, the same reviewer notes the following intriguing fact: although the additive presupposition of *even* should be satisfied if even one of the other (more capable) students passed the test, the *even*-sentence remains quite odd in (36).
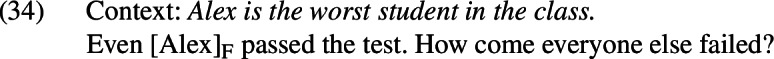


This is puzzling, and it suggests that there may be an additional factor at play. Upon closer inspection, it appears that there is something generally odd about using *even* in examples like these when a relatively small number of the students who took the test passed it—that is, when few of the salient focus alternatives are true. This is illustrated in (37).
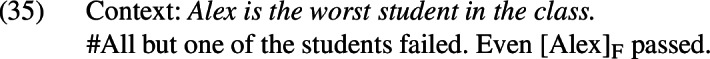


Intriguingly, native speakers express an intuition that it matters whether it was expected that most of the students would pass the test. In the absence of other information, we might assume that when a class takes a test most students will pass it and very few will fail it. If we alter the context to disrupt this expectation,^22^ using *even* when a single non-prejacent alternative is true, as in (38-B), is significantly improved compared to (36). Crucially, *even* remains unacceptable when the prejacent is the only true alternative, as in (38-B). This is exactly the pattern that is predicted if *even* has an additive presupposition.
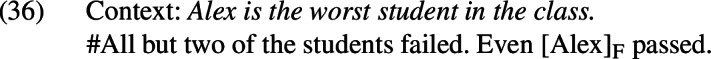


There remains much to be understood about the dispreference for using *even* when, contrary to expectations, only a small number of the relevant alternatives are true; this is a worthy avenue for further investigation, but I will not explore it further here. This phenomenon may reflect a more general pattern of *even* being sensitive to more aspects of the context than is typically acknowledged; see Greenberg (2018b) for discussion of this issue in connection with *even*’s scalar presupposition. For our purposes, it is enough to know that, when this independent constraint is neutralized as in (38), *even* remains unacceptable when the prejacent is the only true alternative.

## Crosslinguistic extensions

The puzzling asymmetry identified in Sect. 1 is not restricted to English; it is reproduced for *even*-like items in Russian (*daže*),^23^ Greek (*kan*), and German (*überhaupt*).
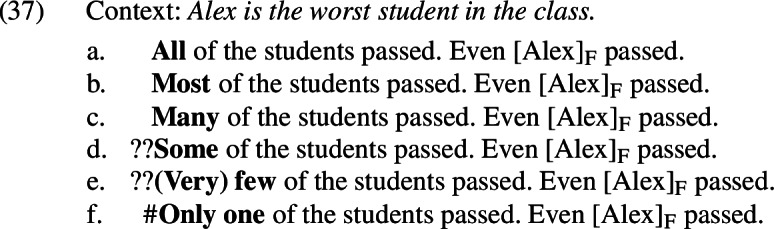


All of these items have an *even*-like scalar component (Iatridou & Tatevosov 2016). *Daže* is the straightforward counterpart of *even* in Russian. *Kan* is one of several *even*-like items in Greek; often translated into English as *so much as*, as shown in (42), it has the distribution of an NPI, meaning that it is restricted to downward-entailing environments (Giannakidou 2007). 
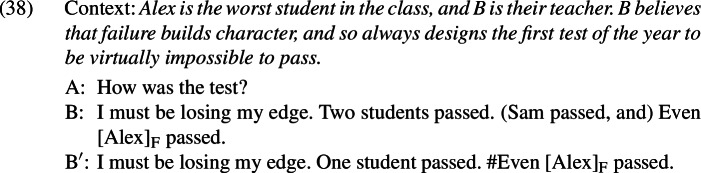


The unacceptability of *kan* in positive presupposition denials is thus unsurprising, but it fits with the crosslinguistic pattern nonetheless.^24^*Überhaupt*, on the other hand, does not mean *even*; variously translated as *absolutely, to a high degree* in positive environments and *at all* in negative environments, as shown in (43), it is characterized by Anderssen (2006) as a generalized domain widener.^25^ What matters for our purposes is that *überhaupt* picks out the strongest or most noteworthy value on a scale, just like *even* does.
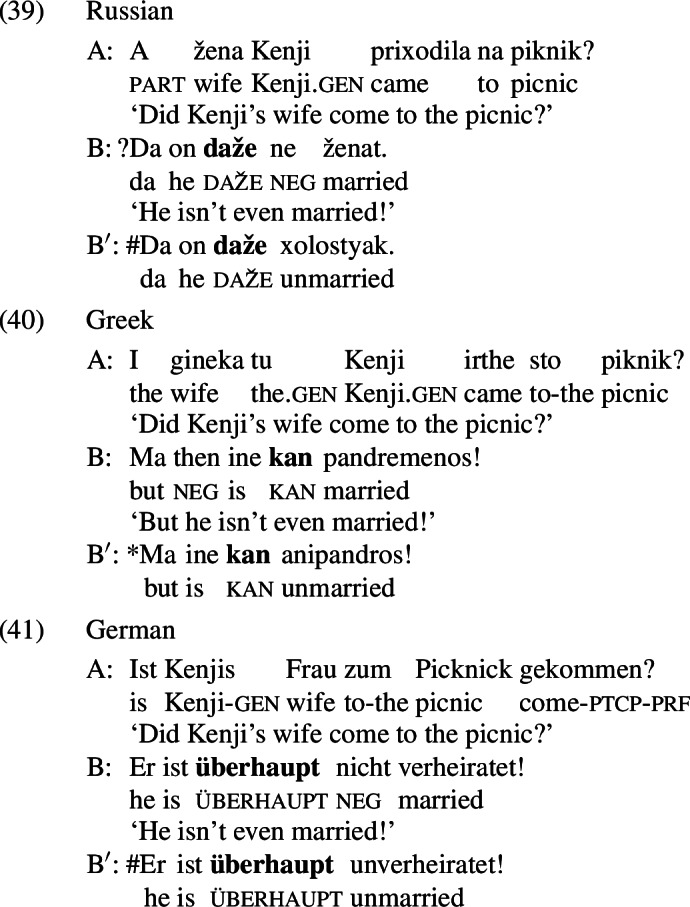


Importantly, these items also have an *even*-like additive component. For example, *daže* and *überhaupt*, like English *even*, are unacceptable in the medals scenario discussed in Sect. 5; this is shown in (44) and (45),^26^ respectively.
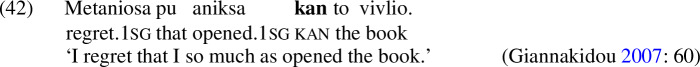


Although Greek *kan* requires a negative environment, it too is unacceptable when its prejacent is the only true alternative; in (46), where Alex is the only individual who Sam did not talk to, *kan* cannot be used.
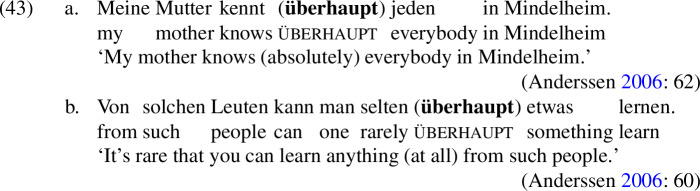


If the contrast between positive and negative presupposition denials with scalar additive particles is due to their additive presupposition, we can make a testable prediction: An item that is scalar like *even* but lacks an additive component will be felicitous in both positive and negative presupposition denials.

I would like to suggest that Hebrew *bixlal* is such an item. *Bixlal* is reported to have an *even*-like scalar component; Greenberg and Khrizman (2012) and Greenberg (2018a) show that *bixlal* has a similar profile to *überhaupt*, as illustrated in (47).^27^
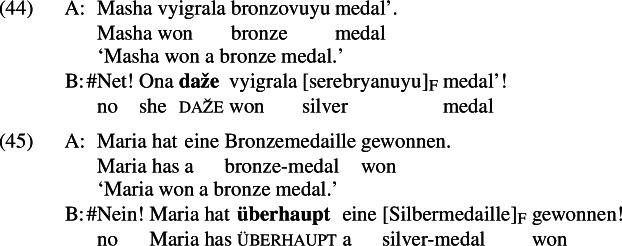


In examples like (48), *bixlal* can be translated straightforwardly into English as *even*. On the scale of places that are difficult to visit from Boston that is made salient in (48),^28^*bixlal* picks out the most noteworthy (i.e., most distant) alternative, as shown in (48-a) and (48-c). It cannot pick out the least noteworthy (i.e., least distant) alternative, as demonstrated by the unacceptability of (48-b) and (48-d). 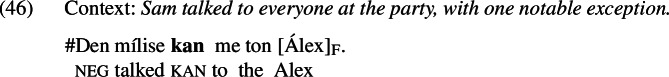


Unlike the *even*-like items we have seen up to this point, *bixlal* is compatible with mutually-exclusive alternatives, as shown in (49); this suggests that it lacks an additive component. 
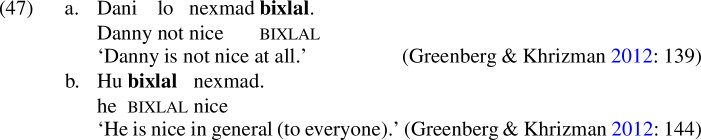


As predicted, *bixlal* can appear in both positive and negative presupposition denials. 
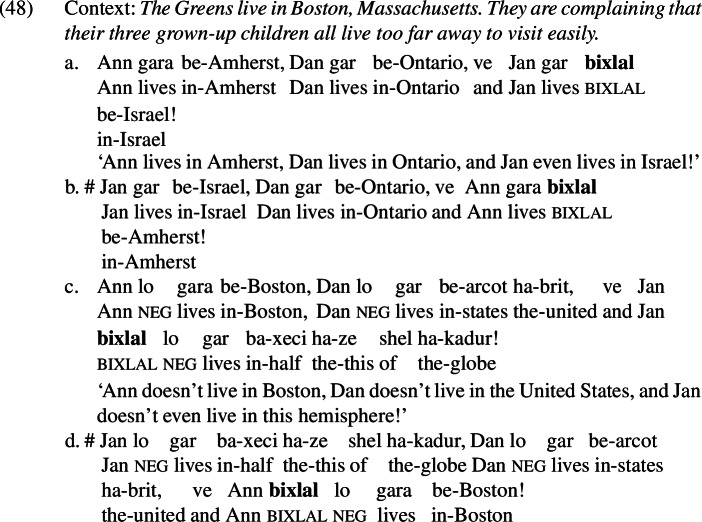


Thus, the crosslinguistic predictions of the proposal appear to be supported. It was the additive presupposition of *even* that was responsible for its infelicity in positive presupposition denials. This section has demonstrated that *even*-like expressions in other languages that share this additive component exhibit the same behaviour, while *even*-like expressions that lack this additive component (i.e., *bixlal*) do not.

## The position of *even*

We have seen that the proposal made in Sect. 3 successfully derives the contrast between positive and negative presupposition denials with *even* in (51), repeated from (2), and makes good crosslinguistic predictions.
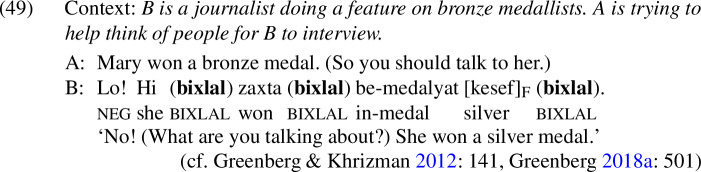


Nothing in the proposal relied on the surface position of *even* with respect to negation; in light of this, the contrast in (52) is surprising.
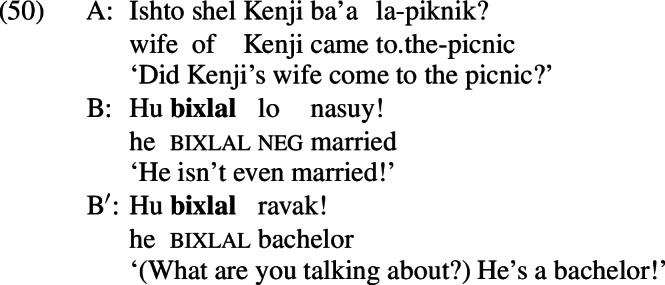


On the scope theory of *even* that we have been assuming, we might expect (53) to be a possible LF for both (52-B) and (52-B). Why then does the surface position of *even* matter?
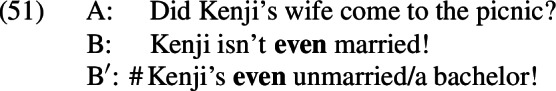


One immediately appealing possibility is that *even* simply cannot occupy the position that it occupies in . Indeed, native speakers of English appear to quite generally disprefer spelling out sentential *even* above negation when it could have been spelled out below negation instead. This is illustrated by the minimal pair in (54), which does not involve presupposition denial.
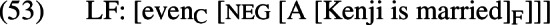


This preference for a low surface position appears to hold with respect to other embedding operators too; this is demonstrated in (55), where the relevant operator is a modal.
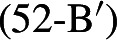


However, there are cases where speakers prefer *even* to be spelled out above an embedding operator. This is shown in (56), where *even* is acceptable above but not below the modal; since *must* surfaces above negation in negative sentences, this means that *even* can in principle be spelled out above negation.
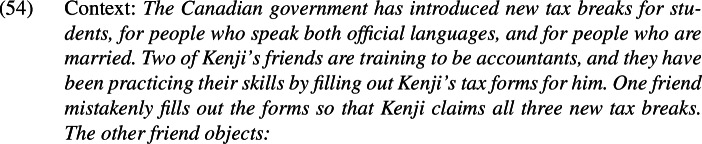


Note that this example differs from the preceding ones in the relative scopes of *even* and its focus associate.^29^*Even* is only able to associate with material that originated in its surface scope (Jackendoff 1972; Erlewine 2014a, b). This means that *even* has different focus association possibilities when it appears above and below negation, as schematized in (57).
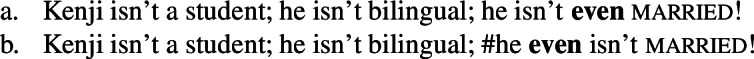
30

These focus associates yield different sets of focus alternatives. Recall that what prevented the negative presupposition denials with *even* from suffering the same fate as the positive ones was the fact that all of the alternatives contained the presupposition-denying [neg A] combination. This combination is guaranteed to be present in (57-a), but not in (57-b), because in the latter case the negation and A-operator are included in the focus associate and thus eligible to undergo substitution. If we were to further suppose that *even* cannot occupy this higher position unless these increased focus association possibilities are going to be exploited, we would have an explanation for the asymmetry. On this view, the problem is not that a parse with an A-operator under negation is not accessible in (52-B’); the problem is that selecting such a parse when these operators are part of what is focused does not guarantee that all of the alternatives will contain these operators.

This pattern is reminiscent of constraints that have independently been proposed for focus-sensitive items in German by Jacobs (1983) and Büring and Hartmann (2001). They propose that the principle in (58) restricts the position of focus particles (FPs) to the lowest eligible attachment site.^31^
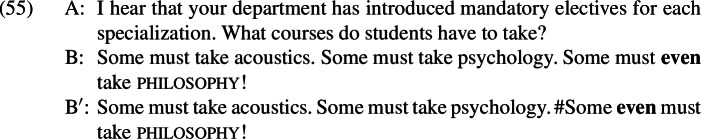


This constraint is embedded within an account that takes all German focus particles to be sentential adverbs, even when they appear to adjoin to sub-clausal constituents. When combined with other principles governing focus particle placement (e.g., that the particle must c-command its associated focus), it amounts to a requirement that focus-sensitive items attach to the lowest clausal node above their focus associate. However, the application of this constraint is sensitive to meaning; Closeness may be violated in order to derive a different meaning.

The proposal that focus particles always attach to the clausal spine in German is controversial (see, e.g., Reis 2005; Smeets & Wagner 2016), and I will not attempt to extend it to English. Instead, it is useful to maintain a distinction between those focus particles that adjoin to clausal constituents (sentential focus particles) and those that adjoin to subclausal constituents (constituent focus particles).^32^ The pattern in (52) can then be captured by the principle in (59).
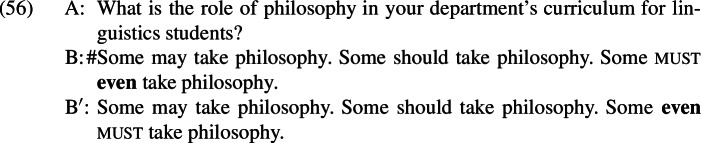


Note that this condition only concerns sentential operator positions; constituent operator positions are not included in the calculation of the closest position to the focus. Thus, the availability of the DP-adjacent position for *even* in (60-a) does not block the availability of the vP-adjacent position in (60-b) but the vP-adjacent position does block the NegP-adjacent position in (60-c).
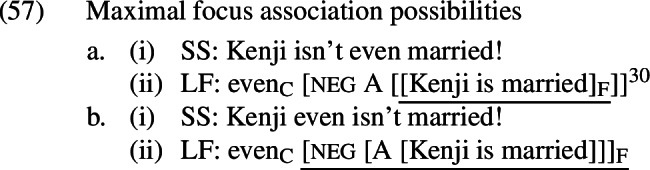


The relativization to meaning allows us to capture an important difference between *even* and *only*: whereas both positions in (60-b) and (60-c) yield the same meaning and so are competitors for one another, (61-b) and (61-c) do not, and so are not.
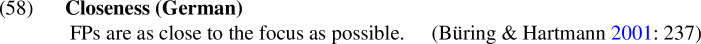


I believe that this is the correct analysis, but before closing this subsection I would like to raise and ultimately reject another possible explanation.

One might imagine that the contrast in (52) reflects a closer link between the *even* and the A-operator than we have been assuming up to this point. There is precedent for an idea along these lines in the literature; for reasons independent of presupposition denial, Erlewine (2014b) builds an A-operator into the scalar presupposition of *even*. More particularly, he proposes that the scalar presupposition of *even* does not rank *even*’s alternatives directly but rather ranks the propositions resulting from applying the A-operator to *even*’s alternatives. Erlewine crucially assumes the ambiguity theory of *even*, which would mean that *even* – and the A-operator in its scalar presupposition – would be interpreted in different places in (52). Although Erlewine does not discuss the additive presupposition of *even*, a straightforward extension of his account to include an A-operator in the calculation of *even*’s additive presupposition would allow us to capture the contrast in (52). When *even* is spelled out below negation, as in (52-B), both *even* and the A-operator will be interpreted under negation. The latter will yield the presupposition-negating configuration we require. When *even* is spelled out above negation as in (52-B’), the A-operator will be interpreted above negation. Just as in the positive presupposition denials, in the absence of a (second) higher negation, this will result in an unsatisfied presupposition being asserted in the non-prejacent alternatives, making them false and thus unable to satisfy *even*’s additive presupposition.
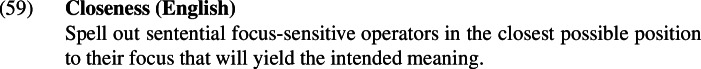


On this view, the position of interpretation for the A-operator would track the surface position of *even*. The two sentences in (52) would thus crucially differ in whether the A-operator is interpreted above or below negation. As we have seen, having an A-operator under negation is necessary to ensure the definedness of the non-prejacent alternatives so that they can satisfy *even*’s additive presupposition; on this view, we would therefore predict only the lower spellout of *even* to be acceptable.

The analysis just sketched would require significant auxiliary assumptions in order to work. Firstly, having *even* and an A-operator packaged together is not on its own enough to ensure the unacceptability of (52-B’); we would need to strengthen this assumption by ruling out introducing an A-operator another way. Yet it does not seem desirable to say that the only way an A-operator can be introduced is as part of the meaning of *even*; there are cases of presupposition denial under negation, like (21), that do not contain *even*. This story would also fail to capture data beyond English: although English *even* (and Greek *kan*) must be spelled out below negation in presupposition denials, Russian *daže* and German *überhaupt* are spelled out above negation. This is shown in (64) and (65), repeated from (39) and (41), respectively.
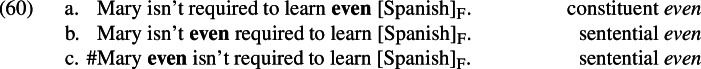


Systematically tying the position of the A-operator to the surface position of scalar additive items would wrongly predict the presupposition denials in (64) and (65) to be infelicitous. To capture the German and Russian data, we must allow A-operators to be inserted independent of the surface position of any scalar additive operators in the structure.^33^ Once we allow this mechanism into the grammar for German and Russian, I see no reason to not do so for English as well.

## What is *even* even doing in presupposition denials?

At this point in our investigation it may be useful to consider what *even* contributes to the presupposition denials where it is felicitous. Because the entailment relation between the prejacent and the alternatives in negative presupposition denials results in both of *even*’s presuppositions being trivially satisfied, *even* should not be contributing any informative inference. Yet the presence of *even* (and its crosslinguistic relatives) has an effect; presupposition denials that contain these items are felt to express a stronger objection than those that lack these items. Furthermore, all of the items that we have seen play this role share a scalar semantics. Is this a coincidence, or is there some deeper connection between scalarity and presupposition denial that the proposed account is missing?^34^

We might start by asking why it feels so natural to embellish a perfectly serviceable presupposition denial like (66-B) with additional material, as in (66-B’).
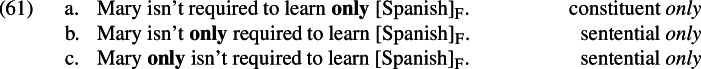


This question could just as well be asked about the presupposition denials in (67).
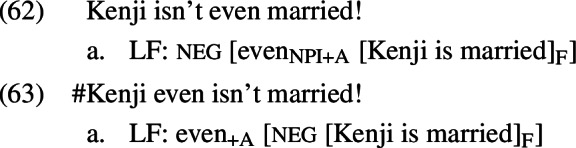


Presupposition denials occur when one conversation participant has presupposed something that is not in the common ground and another participant is unable to accommodate that presupposition because they believe it to be false. Under these circumstances, it is necessary for a cooperative speaker to speak up; the common ground has become untenable and cannot be fixed without explicit renegotiation.^35^ While something like (66-B) will do the job, this kind of discourse move is so important that it is worth taking steps to make sure that it does not slip by unnoticed—for example, by adding material to it.

Now we can ask why, if one is going to add something to a presupposition denial, a focus-sensitive operator is a good candidate. The answer is that focus-sensitive operators make salient certain alternatives to what was said. In the case of the denial of the presupposition that Kenji has a wife, we have assumed that the non-prejacent alternatives are the metalinguistic negations of the congruent discourse moves—both of which are entailed by the prejacent. This allows *even*-denials to communicate something similar to (67-B’) (cf. (21)), though differing in the discourse status of the metalinguistic negation piece.

Why is it that, in those presupposition denials that have been embellished with a focus-sensitive operator, that operator so often has a scalar (additive) semantics? The answer appears to be simply that an *even*-like scalar presupposition is compatible with a set of alternatives that are all entailed by the prejacent. Other focus-sensitive operators, like *only* and *also*, have meanings that are not compatible with such alternatives.^36^ On this view, we should not be surprised if we find non-scalar items populating presupposition denials, as long as they have meanings that are compatible with the presupposition-denying discourses we have been considering.

There is one special effect produced by scalar (additive) operators in the presupposition denials we have been considering that may make them particularly useful additions to these discourse moves. The presence of these items seems to highlight the severity of the breakdown in communication caused by the failure of the presupposition that is being denied, with the result that *even*-denials are felt to be rather impolite; they seem to suggest that the interlocutor’s mistake in presupposing whatever is denied is particularly egregious, something whose consequences they should have foreseen. This effect appears to be a by-product of having *even* apply to a set of alternatives that are entailed by the prejacent, and it is not restricted to presupposition denials like (68). The same feeling arises in (69).
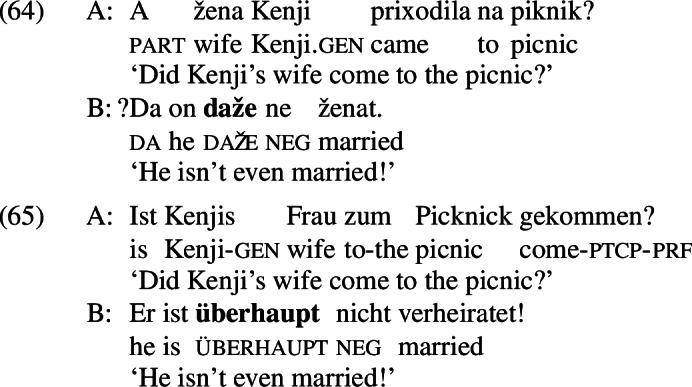


I suspect that this effect emerges precisely because the scalar component of *even* is trivially satisfied. The scalar presupposition of *even* makes explicit that being married is a precondition for having a wife who does or does not attend a picnic, and that reading at least one book is a precondition for reading ten. Being so explicit about something that a competent speaker should already know can be viewed as a face-threatening act. Other scalar items could in principle have subtly different effects depending on their particular semantics. Indeed, native Hebrew speakers report that *bixlal* is only acceptable in objections to the extent that it changes the question under discussion—for example, by addressing a more basic question than the one that the interlocutor has raised, as is the case here. This is similar to what Rojas-Esponda (2014) claims for *überhaupt*, although it is not clear how the observed asymmetry in presupposition denials would be derived on the semantics she proposes. Although these particles add flavour to the presupposition denials in which they occur, their presence is not necessary for presupposition denial to be achieved.

## Loose ends and further extensions

Before concluding this paper, I would like to explore further predictions of the proposal, as well as some possibilities for extending the proposal to other kinds of objections to discourse moves.

### Irreparable infelicity

The proposal derives the infelicity of positive presupposition denials with *even* from a failure of *even*’s additive presupposition. We should therefore predict that the positive sentences would become acceptable if we could supply them with a true alternative.

To see how this might be done, we will need to be explicit about our assumptions regarding how C, the set of salient alternatives that *even* accesses, is built. As is standard in Roothean approaches to focus, we have assumed that these alternatives are derived from the prejacent by making substitutions within the focused part of the sentence that hosts *even*. Following Fox and Katzir (2011), there are three ways of being an eligible substitution: (i) being an element of the lexicon, (ii) being a subtree of the prejacent, or (iii) being a contextually salient constituent. We exploited option (iii), arguing that what is substituted is a proposition made salient by the addressee’s discourse move. These propositions all contained the trigger for the presupposition denied by the prejacent. Although some of the propositions that were salient in the discourse contained negation, none of them contained an A-operator (i.e., the A-operator was not part of the substitution source); this made it impossible to produce the desired presupposition-negating configuration neg > A > $\phi$ in any of the alternatives for the positive sentence with *even*. It is this configuration that ensures that, when a presupposition-bearing proposition $\psi$ is substituted for $\phi$, its presupposition will be part of what is negated, yielding a true proposition that satisfies the additive presupposition of *even*. In negative sentences this configuration can be produced no matter what substitutions are made, because both the A-operator and negation are outside of the focused constituent. This is shown schematically in (70).
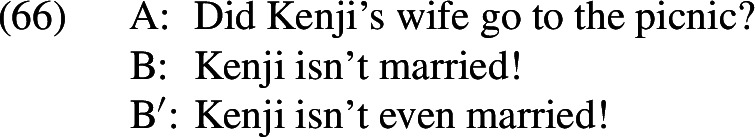


This allows us to make a prediction: If we could supply *even* with an alternative that contains the A-operator under negation, positive presupposition denials with *even* should become acceptable. The relevant examples are given in (71), where the familiar presupposition-denying *even* sentence is preceded by a negated sentence carrying the presupposition trigger—a sentence that can only be acceptable if it is parsed with an A-operator under negation (cf. (21)). Because the string *[* neg *[A [Kenji’s wife came to the picnic]]]* has just been uttered, it will be salient and thus part of the substitution source. This makes it possible for *even* to access a true alternative, shown in bold in (72).
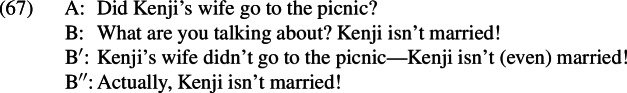


Surprisingly, native speakers judge these responses to be just as unacceptable as the original positive examples. We can confirm that the source of the unacceptability is not difficulty accessing a presupposition-denying parse for the first clause (i.e., one that includes an A-operator below negation) because there is a clear contrast between the responses in (71) and those in (73). If such a parse were unavailable the continuations with *even* would be false, yielding incongruity in (73).
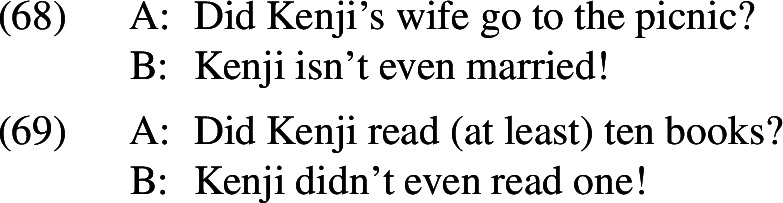


I do not know why this is the case. The impossibility of repairing positive presupposition denials with *even* suggests that we may need to make changes to the proposed analysis of presupposition denials with *even*, the mechanism by which alternatives are formed from the prejacent, or both. I will leave the task of determining which of these options is most appropriate to future work.

### Variation among triggers

In the initial presentation of the puzzle, we saw that negative sentences containing *even* can be used to deny the presuppositions of a variety of triggers, including the existential presupposition of the possessive, the additive presupposition triggered by *again*, and the change-of-state presupposition of predicates like *quit* and *open*.
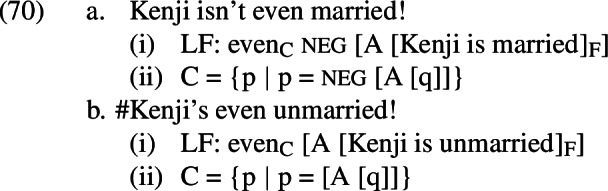


However, not all presuppositions are equally targetable by an *even*-denial. The judgements here are delicate and, unlike for (74)–(76), vary across speakers. For example, most but not all of the speakers that I consulted accept *even*-denials of the presuppositions of emotive and cognitive factive predicates. The relevant examples are given in (77) and (78), respectively.
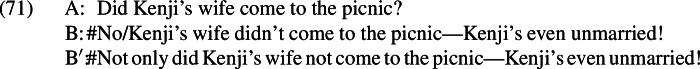


Judgements are similarly variable for *even*-denials of gender presuppositions, exemplified in (79), and uniqueness presuppositions, exemplified in (80); while some speakers accept these examples, many find them to be infelicitous.
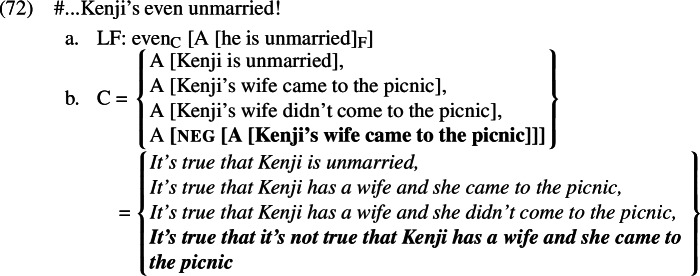


The analysis proposed above cannot, on its own, account for this variation; the A-operator should treat all presupposition failures identically, and so we should expect all of these examples to be just as felicitous as (74). At the same time, the observation that the presuppositions of different triggers do not behave uniformly in all respects is not new; several classification systems have been proposed to divide up these triggers in different ways, with the goal of capturing their varying behaviour (see, e.g., Zeevat 1992; Abusch 2002; Sudo 2012; Sauerland 2013). Some of these classifications have been explicitly tied to how the presuppositions of different triggers behave in focus alternatives. For example, Walker (2012) proposes that, in the terminology of Abusch (2002), the presuppositions of soft triggers do not arise in focus alternatives, whereas those of hard triggers do. Sauerland (2013), on the other hand, proposes that the relevant distinction is between what he terms purely presuppositional triggers, which are truth-conditionally vacuous, and non-purely presuppositional triggers; he claims that the presuppositions of the former are not triggered within focus alternatives while those of the latter are.

Interestingly, the availability of *even*-denials does not appear to track known classes of presupposition triggers. For example, the soft vs. hard distinction would predict that prestate presuppositions, cognitive factive presuppositions, and uniqueness presuppositions would pattern together to the exclusion of existence presuppositions, emotive factive presuppositions, and gender presuppositions. The purely vs. non-purely presuppositional distinction, on the other hand, would predict that the additive presupposition of *again* patterns with gender and uniqueness presuppositions to the exclusion of the others. Neither of these is the pattern observed in the examples above, as summarized in Table 1.

**Table 1:** Variation in availability of *even*-denials across triggers in English

Presupposition	*Even*-denial?	Soft trigger?	Purely presup. trigger?
existence (*’s*)	Yes	No	No
additive (*again*)	Yes	No	Yes
prestate (*open*)	Yes	Yes	No
cognitive factive (*know*)	%	Yes	No
emotive factive (*be sad*)	%	No	No
gender (*she*)	%	No	Yes
uniqueness (*the*)	%	Yes	Yes

I do not know why the acceptability of *even*-denials varies across triggers in this way. Furthermore, preliminary investigation suggests that the set of presuppositions that can be objected to with a sentence containing a scalar (additive) item is not the same in all languages.^37^ Whether this reflects differences in the fine-grained semantics of the particles involved, differences between the relevant presupposition triggers, or something else entirely, is at present unclear. The findings discussed in this subsection raise important questions about what property differs between those triggers whose presuppositions can be denied with *even*-like items and those whose presuppositions cannot, and in what ways this can vary across languages and speakers. While I will not pursue answers to these questions here, it appears that presupposition denials offer a useful testing ground for investigating variation among presupposition triggers.

### Beyond lexically triggered presuppositions

The account proposed in Sect. 3 derives the asymmetry observed in Sect. 1 from a combination of the semantics of *even* and the properties of presupposition denial. In this subsection, we will examine two apparent instances of the asymmetry that do not obviously involve the denial of any lexically triggered presuppositions:
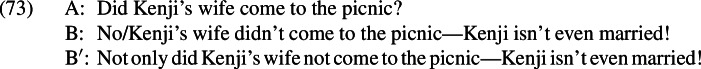


In these dialogues, as in the cases we have considered up to this point, a sentence containing *even* objects that some necessary precondition for Speaker A’s discourse move to be felicitous does not hold. The goal of this subsection is to determine whether we should revise our analysis to capture these cases.

In (81), it turns out that the similarity to our puzzle is only apparent. When talking about test scores, *ace*, *pass*, and *(not) fail* are scalar alternatives of each other, related by entailment. This logical relation encourages a parse where focus is restricted to the verb, as in (83)–(84):
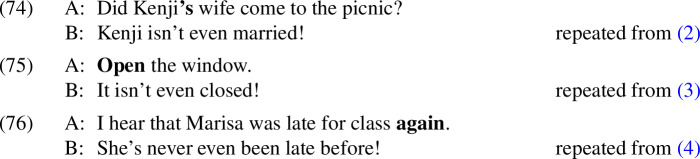


In (83), one of the non-prejacent alternatives (namely, *He didn’t ace it*) is entailed by the prejacent and so will be true whenever the prejacent is true; this guarantees that the additive presupposition of *even* is satisfied. The scalar presupposition of *even* will be satisfied just in case it is less likely that Dan didn’t pass the test than that he didn’t fail it—that is, just in case it is less likely that he failed than that he passed;^38^ this is compatible with a scenario where Dan is known to be a reasonably competent student. In (84), however, both of the non-prejacent alternatives are false when the prejacent is true, meaning that the additive presupposition cannot be satisfied. In this way, we can derive the asymmetry without appealing to A-operators or presuppositions in the alternatives.

It is worth noting that this explanation relies on the focus being restricted to the verb in these cases. If *even* focused a proposition-sized constituent, as argued above for the clear presupposition denials, we would instead expect the following alternatives to be available when these sentences are uttered in response to the polar question *Did Dan ace the test?*:
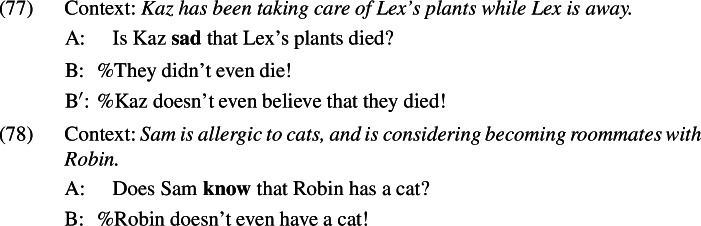


In contrast to the first parse we considered, here *even* has access to a true alternative in the positive sentence, namely *He didn’t ace it*. This is entailed by the prejacent, and so the additive presupposition is predicted to be satisfied, yielding felicity. Why then is this parse not selected over the infelicity-yielding one? One possibility is that this parse is ruled out by a general pressure to keep focus marking minimal in the grammar, such as Schwarzschild’s (1999) AvoidF constraint. A second possibility is that the parse is not ruled out but the relevant alternative is difficult to access. The alternative that satisfies the additive presupposition for the positive sentence corresponds to the negative answer to the polar question (*He didn’t ace it*). There is evidence that negative answers are quite generally more difficult to retrieve from the context than positive answers when they are not mentioned explicitly.^39^ Consider the following minimal pair, due to Sabine Iatridou (p.c.):
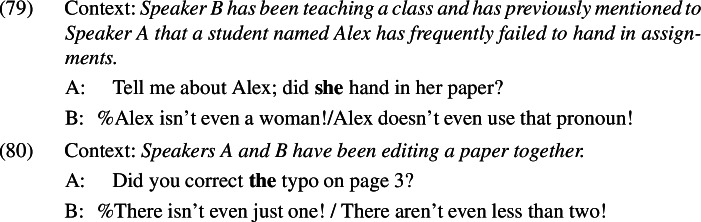


These examples differ with respect to which of the polar question’s answers must be accommodated in order for the continuation to make sense. In (87-a), the positive answer (*Yes, I want to live a long life*) is easily accommodated; in (87-b), however, the roughly equivalent negative answer (*No, I don’t want to die young*) is much more difficult to retrieve than the incongruent positive answer, yielding oddness.

Returning to the example in (81), directly supplying *even* with the relevant true alternative as in (88) makes the positive *even* sentence felicitous.
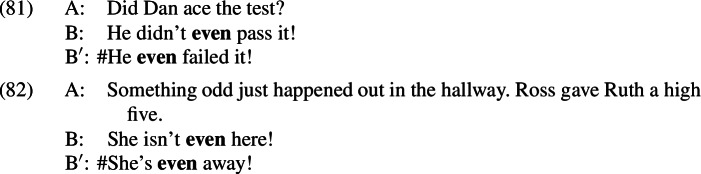


This suggests that the positive sentence in (81) is only unacceptable to the extent that it is difficult to access i) a parse and ii) the relevant alternative that will allow the additive presupposition of *even* to be satisfied.^40^ This makes (81) crucially different from the clearly presupposition-denying uses of *even*, which could not be salvaged even when supplied with an appropriate true alternative.

In (82) the situation is more complex. Unlike the previous case, there is no motivation to assume narrow focus. Following the strategy pursued in Sect. 3 and assuming the LFs and sets of alternatives in (89) and (90) leaves the asymmetry unexplained. On the reasonable assumption that Ruth being absent precludes her being the recipient of a high five from Ross, both sentences have an alternative that is contextually entailed by the prejacent, namely *Ross didn’t give Ruth a high five*. *Even*’s additive presupposition will thus be satisfied in both cases, while its scalar presupposition will in both cases require that the prejacent is less likely than the non-entailed alternative (that Ross gave Ruth a high five); the difference in their felicity is therefore unexpected.
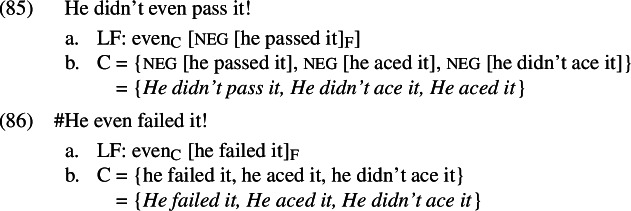


Unlike example (81), but like the presupposition denials discussed in Sect. 3, the positive sentence is not improved by making the true (negative) alternative overt.
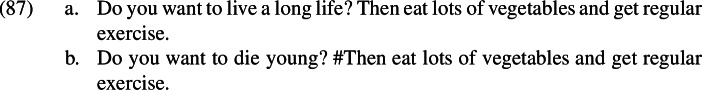


Intuitively, what the *even* sentences do here is deny that it was possible to give Ruth a high five (on the grounds that Ruth is not here), with this being a necessary precondition for the truth of Speaker A’s assertion. Unlike in the cases dealt with in Sect. 3, what is denied by the prejacent does not follow equally from both of the alternatives; the alternative *Ross didn’t give Ruth a high five* does not obviously entail or presuppose that it was possible for him to do so. The lack of incompatibility between the prejacent and this alternative is what makes it possible for the additive presupposition to be satisfied in both the positive and the negative *even* sentences. To derive the unacceptability of the positive *even* sentence, we need to exclude the true proposition *Ross didn’t give Ruth a high five* from the set of alternatives that *even* has access to in this case. If we could replace this alternative with something like *It was possible for Ross to give Ruth a high five but he didn’t do so,* we would have a situation exactly like the cases we have successfully dealt with.

We could in principle encode the possibility requirement as a presupposition. If *Ross gave Ruth a high five* and *Ross didn’t give Ruth a high five* presupposed that it was possible for him to do so,^41^ we could import our analysis of presupposition denials directly from Sect. 3 to derive the asymmetry. This felicity condition has an intuitive appeal; if something is not possible, it is odd to deny that it occurred. Dynamically speaking, if the context set contains no worlds where *p* is true, asserting $\lnot$*p* is vacuous because there are no *p*-worlds to rule out. Perhaps, then, we could understand this presupposition as a way of banning vacuous discourse moves. One might well object that this kind of general pragmatic principle should not be encoded as a presupposition; nevertheless, something along these lines would get us the right results for the case at hand.

We could achieve an equivalent result by modifying our assumptions about what propositions Speaker A’s discourse move makes salient. Stephenson (2005) suggests that, in the absence of contrastive focus, the default alternatives that a sentence *p* makes salient are *p* itself and the proposition that *p* failed to obtain, which she takes to be not the simple negation of *p* but rather the negation of *p* conjoined with the claim that *p* was possible. This suggestion is situated in a discussion of quantificational readings of indefinites (cf. Diesing 1992), but let us suppose that it can be extended to sentences that do not contain indefinites. Taking a declarative sentence to make salient these propositions would directly supply the desired alternatives in (92)–(93).
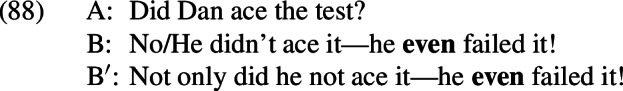


The true alternative that made us predict acceptability for the positive sentence (*Ross didn’t give Ruth a high five*) is no longer present in (93-b); it has been replaced by *Ross could have given Ruth a high five and he didn’t do so*. This alternative will be false when the prejacent is true, because the prejacent contextually entails that Ross could not have given Ruth a high five.

I will not attempt to decide between these two approaches here. In different ways, they both remove the problematic alternative *Ross didn’t give Ruth a high five* from the set of alternatives for the positive sentence and replace it with an alternative that is incompatible with the prejacent.^42^ What matters for our current purposes is that the data in (81) and (82) can be accounted for without altering the core proposal made in Sect. 3.

## Conclusion

This paper has identified a surprising contrast between positive and negative presupposition denials with *even*. It has also provided a new argument in favour of *even*’s additive presupposition: when combined with the alternatives that are salient in the relevant contexts and an independently motivated mechanism that allows presuppositions to be negated, it can explain the behaviour of *even* in presupposition denials.

The account presented here has consequences for how we think about presuppositions generated in alternatives. If the analysis presented here is correct, we pay attention not only to the presuppositions of the sentences that we hear but also, in some cases, to the presuppositions of alternative sentences that we do not hear. Moreover, the variation in the acceptability of *even*-denials laid out in Sect. 9.2 connects the present work with other recent investigations of variation among presupposition triggers and the projection properties of focus alternatives (e.g., von Heusinger 2007; Walker 2012; Sauerland 2013; Mayr & Romoli 2016; Spector & Sudo 2017). What is unique about the puzzle presented here is that this is a case where a presupposition triggered in a focus alternative can have an effect on the acceptability of a sentence that does not itself contain the trigger for that presupposition. This raises important questions for how we think about the status of alternatives in the grammar. Presuppositions are usually understood as definedness conditions that the context must meet in order for a discourse move to have its intended effect. What kinds of constraints can alternatives place on the use of sentences that invoke them? To what degree are these effects dependent on the particular semantics of the operator that generates the alternatives in question? These are questions that merit further investigation.

## Appendix A: The proposal in the ambiguity theory of *even*

This appendix shows how the proposal presented in Sect. 3 can be translated into a lexical ambiguity theory of *even*. While the two theories do come apart in other areas of the grammar, the choice of theory does not matter for the purpose of capturing the data discussed in this paper.

The lexical ambiguity and scope theories of *even* differ only in their treatment of *even* in NPI-licensing environments; this means that we can import the account of the unacceptability of positive presupposition denials with *even* directly into a lexical ambiguity account. Positive presupposition denials with *even* will have the LF and set of alternatives in (94), repeated from (24); because none of the alternatives can be true when the prejacent is true, the additive presupposition of *even* is not satisfied.

All that is left is to derive the acceptability of negative presupposition denials with *even*.

Recall that, in the ambiguity theory, the NPI *even* scopes below negation in negative sentences; this means that our flagship negative example will have the LF in (95-a) and the set of alternatives in (95-b).

Aside from the prejacent, the set of alternatives in (95-b) is identical to the one generated for the positive sentence in (94-b). However, the NPI *even* introduces different presuppositions. Whereas the additive presupposition of the non-NPI *even* requires that there is a non-prejacent alternative that is true, the NPI *even* in (95) requires that there is a non-prejacent alternative that is false. As we have already seen, all of the non-prejacent alternatives in this set are false; the additive presupposition of the NPI *even* will therefore be satisfied in (95). The scalar presupposition will also be trivially satisfied, because the NPI *even* presupposes that its prejacent is the most likely of the alternatives, and the prejacent here is entailed by each of the non-prejacent alternatives. Thus, it is possible to derive the observed asymmetry under a lexical ambiguity theory of *even*; presupposition denials are not a particularly informative data point in the debate between the scope and ambiguity theories of *even*.

A reviewer notes that choosing the ambiguity theory of *even* might complicate the strategy adopted in Sect. 7 to derive the surface position of *even*. The concern here is that, if the two *even*s are separate lexical items, and the non-NPI *even* is not licensed under negation, the lowest eligible spellout position for this *even* in its sentential guise is immediately above negation—precisely the position that we would like to rule out. Intuitively, what is needed is to make the parse with the NPI *even* a competitor to the parse with the non-NPI *even* for the purposes of evaluating Closeness. One way to do this is to understand the Closeness constraint as being indifferent to the identities of the sentential operators in the competitor sentences that it evaluates; it would simply compare any two licit structures containing sentential focus operators that will produce a particular meaning. The meaning restriction will, in most cases, result in the structures being compared containing the same focus operator. However, there are some pairs of items—the two *even*s, but also, for example, the additive particles *either* and *too* (see, e.g., Rullmann 2003)—whose meanings are related in such a way that, when one member of the pair attaches above negation, it will produce a meaning that is identical to the one produced when the other member of the pair attaches below negation. In precisely these cases, Closeness would rule out the former structure in favour of the latter.

One might wonder whether the polarity-based asymmetry we have observed can be derived directly from the alleged ambiguity of *even*, for example by saying that only the NPI *even* is able to participate in presupposition denial. It is not clear why such a restriction would exist, but it could be stipulated; for example, by having the non-NPI *even* presuppose that all of its alternatives are defined and banning a parse of positive presupposition denials that includes the A-operator. The latter could be done by taking the A-operator to itself be an NPI. Importantly, we would still need all of the machinery employed by the proposal in Sect. 3 to account for the acceptability of negative presupposition denials with $even _{\textsc {npi}}$. This NPI *even* will scope below negation, but its alternatives will still contain the trigger for the presupposition being denied; we must therefore still posit that it is accompanied by an A-operator to ensure that the alternative propositions are false rather than presupposition failures, exactly as sketched above.

Reducing the polarity-based asymmetry to polarity sensitivity of *even* would require more stipulations than either the original analysis or its direct translation into the lexical ambiguity framework. Such an account would also lack an explanation for why it is the NPI *even* that is endowed with presupposition-denying abilities; without a principled reason for this, we might expect that other languages might choose the non-NPI *even* as their presupposition denier. In all of the languages examined in Sect. 6 that exhibit an asymmetry between presupposition-challenging uses of particles in positive and negative environments, the asymmetry is in the same direction as English. The fact that the asymmetry discussed in this paper is tied to polarity follows from the mechanics of the A-operator: it can only negate presuppositions when it is under an appropriate negative operator. No appeal to polarity sensitivity is necessary to derive the attested pattern.

## Appendix B: Selecting a smaller focus associate

This appendix addresses the assumption about the size of the focused constituent that was made in Sect. 3. Although for the purposes of capturing the data it is sufficient to show that there is a parse of the relevant sentences that will yield the attested asymmetry, a reviewer wonders whether this is the only parse that will produce this result. More particularly, what would have happened if we had assumed that *even* associates with the VP, as in (96-a), instead of the vP as in (96-b)?

If we were to make this assumption, the focus alternatives that *even* operates on would be formed from the prejacent by making substitutions for the VP (*married/unmarried*). Which properties of individuals will be salient in the presupposition denial contexts that we are interested in? At a minimum, we should expect *unmarried* to be salient and relevant whenever *married* is, and vice versa. On this view, the minimal set of alternatives that *even* considers should be as in (97-b) and (98-b).

In both cases, the scalar presupposition will be contingent but satisfiable; it simply requires that it is less likely that Kenji is not married (i.e., is unmarried) than that he is married (i.e., not unmarried). However, in both cases the additive presupposition will be unsatisfiable because the alternatives are incompatible with each other. If these are the only substitutions available, we would thus fail to predict the acceptability of negative presupposition denials with *even*.

We could, of course, consider additional substitutions. For example, *engaged, dating,* and *single* are properties that are relevant to Kenji’s marital status, since each of these entails being unmarried (under the assumption that being married to someone precludes not only being single but also dating or being engaged to someone else). If we add the alternatives formed by making these substitutions to the sets above, the result will be as in (99)–(100).

The additional alternatives ensure that the additive presupposition will now be satisfied in both (99) and (100). In (100), the additive presupposition will be satisfied as long as Kenji is engaged, dating, or single; under the assumption that these exhaust the possibilities for being unmarried, this condition is guaranteed to be met whenever the prejacent is true. In (99), the additive presupposition requires that Kenji is either not engaged or not dating or not single; under the assumption that one can only be one of single, dating, or engaged at a time, two of these alternatives are guaranteed to be true whenever the prejacent is true.

Matters are different with the scalar presupposition. In (100), the scalar presupposition cannot be satisfied, because each of the new alternatives entails the prejacent. We therefore correctly predict the infelicity of the positive presupposition denial, but this is due to a failure of the scalar presupposition instead of the additive presupposition. In (99), the scalar presupposition is satisfiable but contingent on the context; since Kenji being married (i.e., not unmarried) entails each of the new alternatives, the scalar presupposition will reduce to a requirement that it is less likely that Kenji is not married than that he is married. Thus, under an analysis where *even* associates with just the VP, we predict that a positive presupposition denial like *Kenji’s even unmarried!* will never be felicitous and that a negative presupposition denial like *Kenji isn’t even married!* will be felicitous just in case it is less likely that Kenji is unmarried than that he is married.

Thus, we can derive the attested asymmetry with VP focus, but such an analysis has three undesirable features. Firstly, since in the negative cases the scalar presupposition of *even* is contingent on the likelihood of Kenji being unmarried vs. married, we predict that in contexts that do not satisfy this condition negative presupposition denials will be just as infelicitous as positive ones. This prediction does not appear to be borne out; native speakers report that *Kenji isn’t even married!* is felicitous regardless of the contextual likelihood of Kenji being unmarried vs. married. Secondly, since on this view it is the scalar presupposition rather than the additive presupposition that is unsatisfiable in the positive case, we lose our explanation of the crosslinguistic data; since Hebrew *bixlal* is like the other items investigated in its scalar component, we wrongly predict the asymmetry to be present for this item as well. Finally, it is not clear that the set of alternatives that we would need to assume in order for this analysis to work is going to be salient in the discourse contexts that we are interested in. Following the logic in Sect. 4, the values assumed for C in (99) and (100) are not relevant to the question made salient by A’s preceding discourse move. More particularly, the *engaged, dating*, and *single* alternatives that were needed to satisfy the additive presupposition do not provide (partial) answers to A’s question, since they do not rule out either of the cells in the partition; instead, they draw unnecessary distinctions among the worlds where Kenji isn’t married.

## Footnotes

1 In the cases that we will be discussing, *even* must appear below negation in the surface string. This positional restriction will be discussed in Sect. 7, where I will argue that it is due to independent constraints on the positions of focus-sensitive operators.

2 Like other focus-sensitive operators, *even* does not always attach to obviously propositional constituents; nevertheless, I will assume that *even* always operates on propositions. For a recent defence of this view over a type-flexible approach to such operators, see Hirsch (2017), cf. Wagner (2006, 2013).

3 For more details about what constitutes an eligible substitution, see Sect. 9.1.

4 The quantificational force of *even*’s presuppositions and the precise flavour of its scale are topics of debate in the literature. Nothing hinges on the particular choices made here.

5 The status of the existential presupposition varies in different versions of the ambiguity theory; Rooth (1985: 153) assumes that both versions of *even* include this meaning component, while Rullmann (1997: 58-61) argues that neither version does. The existence of this presupposition is addressed in Sect. 5.

6 A reviewer expresses skepticism that proposition-denoting expressions like vPs can be focused. However, the Roothean approach to focus adopted here does not restrict what kinds of constituents can be focused; in the absence of a principled reason to do otherwise, I will therefore assume that foci of propositional type are possible. Precedent for the idea that focus particles in this position can associate with a constituent that contains both the subject and the predicate can be found at least as far back as McCawley (1970: 296). An illustrative example is provided in (i); in this context, the use of *even* in (i-d) is most naturally interpreted as comparing the likelihood of Harvard holding a pep rally to the likelihood of rain occurring every Thursday at the same time and the likelihood of Sue deciding to be nice to the speaker. Because (i-b) and (i-c) have no material in common with the prejacent of *even* in (i-d), the only way that they can be the relevant focus alternatives for (i-d) is to have all of the material in (i-d) be focused.

7 I will actually assume something stronger: that these are the only substitutions that make it into the contextually salient set of alternatives. More on this later.

8 The presence of the negative proposition (*Kenji’s wife didn’t come to the picnic*) in (16-b) and (17-b) is not crucial to the proposal; it would work just as well if the only proposition made salient by a declarative was the one denoted by the sentence that is uttered, and the only proposition made salient by a polar question is the one denoted by the root of the question.

9 For discussion of this issue, see Erlewine (2014b). He argues for a mechanism that could be extended to yield much the same effect as what I propose below, although it is packaged somewhat differently; the relevant properties of Erlewine’s account will be addressed in Sect. 7.

10 Trivalent systems have three truth values: 1, 0, and # (undefined). The third truth value is assigned to presupposition failures.

11 I formulate the proposal using A-operators because, as we will see, it is important that the presuppositions are accommodated at the same position across the alternatives. Tying this operation to a syntactically represented operator provides a convenient way to enforce this with structurally defined alternatives. I believe that other formal tools for dealing with data like (21) that share this property, such as metalinguistic negation (Horn 1985, 1989), would also work, but something more would need to be said to appropriately restrict local accommodation in these cases if it is not tied to a lexical item (as in Heim ’s (1983) original local accommodation operation).

12 It should be noted that the presence of the A-operator does not result in denial of the presupposition triggered by the definite description *the picnic*. Romoli (2011) argues on independent grounds that the A-operator needs to be able to selectively accommodate some presuppositions in its scope while leaving others untouched. Fox (2013) sketches a way of implementing this idea by co-indexing the A-operator with the trigger(s) of the presupposition(s) it asserts; I direct the interested reader to that work for details.

13 A reviewer asks whether the unacceptability of positive presupposition denials with *even* could be derived without reference to the additive presupposition, on the assumption that *even*’s scalar presupposition is conditionalized on an information state compatible with the speaker’s beliefs; as B believes that it is maximally unlikely that Kenji has a wife, the prejacent of *even* would be the most likely alternative, resulting in a failure of the scalar presupposition. It appears that *even*’s scalar presupposition cannot be restricted in this way when the speaker’s beliefs are known to conflict with the beliefs of their interlocutor. Otherwise, we would expect B’s use of *even* in (ii) to be felicitous.  Note that there is nothing wrong with adopting a likelihood ranking that one’s interlocutor has committed to, even if it conflicts with one’s private beliefs; this is demonstrated in (iii).

14 I abstract away from the possibility that Kenji is married to a man here; strictly speaking, the prejacent corresponds to the subset of $\lnot$q worlds where Kenji has no spouse of either gender.

15 It should be noted that, in later work, Crnič (2013) assumes that the additive presupposition is active when alternatives are mutually exclusive.

16 Note that the judgement persists even if *silver* is replaced by the most difficult-to-achieve medal colour, *gold*; this tells us that the oddness of (iii) is not due to a failure of the scalar presupposition.

17 I believe that this is corroborated prosodically; the subject cannot be deaccented, even in a discourse where John has previously been mentioned.

18 The proper name has been replaced with a pronoun because pronouns, unlike proper names, do not carry a pitch accent unless they are stressed. This makes it easier to tell whether the subject is focused.

19 If there are speakers who find these sentences acceptable in contexts where the alternatives are truly mutually exclusive, they are predicted to accept both positive and negative presupposition denials with *even*.

20 Wagner (2013) is sometimes taken to show that *even* is only additive when it appears next to a nominal expression. However, Wagner takes “additive” to mean something different from how the term is used here. For him, additive operators (including *also*) require both that i) there is a non-prejacent alternative that is true, and ii) that alternative is not in an entailment relationship with the prejacent. In this paper, additivity refers to only the first of these requirements. Wagner correctly shows that this strong additive requirement (i + ii) is only present for *even* when it appears next to a nominal expression, but he does not show that the weaker additive presupposition (i) is absent from vP-peripheral instances of *even* like those discussed in this paper. If vP-peripheral *even* did not have this weaker additive presupposition, we would expect it to be felicitous in an example like the following, contrary to fact.

21 This example bears a resemblance to what are sometimes called degree-based uses of *even*. Zhang (2022) notes that, while such examples do not appear to satisfy an individual-based notion of additivity, they do satisfy a degree-based notion of additivity. To capture these cases, Zhang proposes that *even* presupposes the presence of a salient QUD about degrees; it is not clear how this account would capture the felicity of *even* in the presupposition denials under investigation here.

22 I am grateful to Dan Goodhue for useful discussion on this point, and for his help in constructing this example.

23 Native speakers of Russian report that *voobšče*, an *even*-like item similar to *überhaupt*, can be used instead of *daže*, but the precise judgements vary considerably. Some speakers can use *voobšče* on its own and report a contrast in the same direction as the other languages examined here; others can use this item on its own and report no contrast. There are also speakers who require that *voobšče* be accompanied by the particle*-to*; when*-to* is present, both positive and negative presupposition denials are acceptable for all speakers.

24 Crnič (2011) decomposes *kan* into two morphological components, one of which has the same meaning as *even* and moves covertly over NPI licensers, just as *even* does on the scope theory.

25 Domain widening is only licensed when it yields a stronger meaning. Kadmon and Landman (1993) proposed this condition to capture the distribution of *any*, which they argue is an existential quantifier lexically specified to take a widened domain. Expanding the domain of an existential quantifier yields a weaker meaning in upward-entailing environments but a stronger meaning in downward-entailing environments, resulting in the restriction of *any* to the latter contexts. Anderssen (2006) notes that on this view *any* involves two ingredients (domain widening and existential quantification) which need not be lexically fused. He proposes that *überhaupt* spells out only the domain widening component, which is free to combine with a variety of expressions. If it combines with an existential quantifier, it is confined to downward-entailing environments, as *any* is, but if it combines with a universal quantifier the strengthening condition results in a restriction to upward-entailing environments. Relevantly for our purposes, the strengthening requirement for domain wideners like *any* has elsewhere been formalized with a covert counterpart of *even* associating with a domain, most notably in the work of Chierchia (2006) (cf. also Lee & Horn 1994 and Crnič 2014). Since *überhaupt* spells out this *even*-like meaning component, we should be able to subsume its behaviour in presupposition denials under the analysis developed for the English examples in Sect. 3.

26 When *überhaupt* itself is stressed, it may receive a different translation than when it is unstressed (Anderssen 2006). The uses relevant for this paper are the unstressed ones.

27 Greenberg and Khrizman (2012) argue that *bixlal* is best understood as a generalized strengthener rather than a generalized domain widener as Anderssen (2006) proposes for *überhaupt*. This distinction need not worry us; it is the strengthening component of domain wideners that resembles the scalar component of *even*.

28 Special thanks to Itai Bassi and Danny Fox for their help in constructing this example. *Amherst* here refers to Amherst, Massachusetts.

29 Native speakers do not seem to judge strings like *Kenji even isn’t married* to be acceptable, regardless of what is focused. I believe that this is related to another fact: some speakers find examples like (55) to be less degraded than (54). Anticipating the solution somewhat, I believe that the reason for this is that modal auxiliaries have a different semantic type than non-modal auxiliaries. There are therefore easy-to-access, semantically contentful substitutions available for the modal when *even* is spelled out high that are not available for non-modal auxiliaries.

30 Although we cannot determine the relative positions of *even* and the A-operator in the surface structure due to the latter’s covert nature, I assume for simplicity that the A-operator is not eligible for inclusion in the focus associate.

31 Rullmann (2003) proposes a similar principle (the Low Attachment Preference) that is not restricted to German but applies only to sentence-final particles and adverbs.

32 Here I borrow Erlewine’s (2017) terminology for this distinction. He proposes that a principle very similar to the one discussed here governs the placement of sentential focus particles in Vietnamese; that constraint differs from Closeness in that it is relativized to phases and insensitive to meaning.

33 While we could in principle posit that *daže* and *überhaupt* are both inserted under negation and then undergo obligatory overt movement to their observed positions above negation, the motivation for this movement would be a mystery.

34 An indirect connection between scalarity and presupposition denial has been drawn by Iatridou and Tatevosov (2016). They observe that the presence of scalar (additive) items in questions can trigger an inference of extreme speaker ignorance, which they argue amounts to a denial of the interlocutor’s presupposition that the speaker was competent to engage with the topic of conversation. Iatridou and Tatevosov (2016) derive the extreme ignorance inference by having scalar presupposition operate at the level of discourse moves and then reasoning about why a question might be unlikely to be asked. The presupposition-challenging effect is entirely parasitic on the extreme ignorance inference on this view. The declarative presupposition denials under discussion here differ from Iatridou and Tatevosov’s (2016) questions in that they involve no inference of extreme ignorance and their presupposition-challenging effect is independent of the presence of *even*. As far as I can tell, any attempt to extend their approach of having *even* operate at the level of discourse moves to the cases under discussion here would fail to capture the polarity-based asymmetry.

35 Possible exception: If the presupposition is truly orthogonal to the continued viability of the conversational goals.

36 See fn. 20 for discussion of a relevant difference between the additive requirements of *also* and *even*.

37 One aspect of the pattern in Table 1 does appear to generalize: existence and prestate presuppositions can be denied by the counterpart of an *even*-denial in all of the languages examined in this paper.

38 Note that the prejacent will always be less likely than the entailed alternative *He didn’t ace it*. Thus, the satisfaction of the scalar presupposition in this case depends only on the relative likelihood of Dan passing vs. failing the test.

39 In the terminology of Inquisitive Semantics, the positive answer is “highlighted” by a positive polar question (Roelofsen & Farkas 2015).

40 This diagnosis is further supported by the fact that the asymmetry also disappears when the additive presupposition of *even* is satisfied by the more easily accommodated positive answer to Speaker A’s question, as shown in (v).

41 I will remain agnostic about where such a presupposition would be triggered.

42 A reviewer notes that a third way of achieving this result would be to revisit our assumption that the negative proposition made salient by Speaker A’s discourse move is an eligible substitution; see footnote 8.
